# Impacts of university lockdown during the coronavirus pandemic on college students’ academic achievement and critical thinking: A longitudinal study

**DOI:** 10.3389/fpsyg.2022.995784

**Published:** 2022-10-26

**Authors:** Xiaojing Lv, Juanjuan Ma, Thomas M. Brinthaupt, Shaochun Zhao, Xuezhu Ren

**Affiliations:** ^1^School of Education, Huazhong University of Science and Technology, Wuhan, China; ^2^Department of Psychology, Middle Tennessee State University, Murfreesboro, TN, United States

**Keywords:** academic achievement, critical thinking skills, critical thinking dispositions, COVID-19, distant learning, learning performance, self-management skills

## Abstract

The outbreak of the coronavirus disease 2019 (COVID-19) has resulted in widespread university lockdown. However, impacts of the university lockdown on the learning and academic development of university students have not been thoroughly investigated. The current study examined college students’ changes of learning outcomes during the COVID-19 lockdown period and clarified what might explain individual differences in students’ learning outcomes after they had learned from home for a whole semester when universities were physically closed due to the COVID-19 pandemic. Data were derived from a longitudinal study examining the development of college students including students’ academic achievement and critical thinking (including both skills and dispositions) before and after the university lockdown. We observed significant decreases in critical thinking skills and dispositions from pre- to post-lockdown. Both perceived academic achievement and critical thinking exhibited greater variability after the lockdown. In addition, students’ readiness for online learning, especially their self-management skills, consistently predicted post-lockdown learning outcomes after controlling for pre-lockdown outcomes and family socioeconomic status (SES). Those who have assumed more responsibilities at home, or who were more vulnerable to emotional distress during the pandemic, performed less well in post-lockdown learning outcomes. These findings call for better management of student learning and development when major changes are required in higher education practices for responding to the ongoing COVID-19 crisis as well as other potential situations.

## Introduction

The outbreak of the coronavirus disease 2019 (COVID-19) has resulted in widespread school shutdowns. More than 180 countries around the world have mandated school closures, leaving, at its peak in April 2020, approximately 1.5 billion children and youth out of school (UNESCO, 2020). Just within higher education in China, all colleges and universities shut down and adopted distance education during the spring semester of 2020, affecting more than 17 million college students (Yao, 2020). The university closures discontinued the classic style of higher education, forcing students to adjust to learning at home, as well as to adopt new learning methods (Gonzalez et al., 2020). These dramatic changes are likely to pose significant disruptions to students’ conventional learning and created several uncertainties which may affect students’ learning outcomes. Concerns have been raised with respect to students’ learning losses and greater variability in their academic skills due to school closures or the temporary lockdown of universities (Kaffenberger, 2021). The current paper, by means of a longitudinal study, evaluated impacts of a university lockdown on students’ learning outcomes represented by academic achievement and critical thinking (including both skills and dispositions), and clarified how and to what extent learning from home during the COVID-19 pandemic affected college students’ learning outcomes.

### Potential effects of university lockdowns on students’ learning outcomes

While school and university lockdowns are deemed necessary to mitigate the spread of the coronavirus, they may carry important consequences for students’ academic development. Many studies have examined effects of school lockdowns or home confinements on K-12 students, suggesting that school lockdowns have produced substantial losses in students’ learning outcomes (Azevedo et al., 2021; Clark et al., 2021; Maldonado and De Witte, 2021; Parolin and Lee, 2021). Modeling estimates suggest that these learning losses continue to accumulate even after children return to schools (Kuhfeld et al., 2020). However, at the moment it remains unclear whether the university lockdown affects college students learning performance. Young adults are assumed to experience less difficulty when adapting to distant learning than children, given that most young adults have some online learning experiences while K-12 students rely heavily on classroom learning. Moreover, compared to school-aged children, young adults have acquired some amount of self-management skills to facilitate learning beyond classrooms (Anthonysamy et al., 2020). For example, Gonzalez et al. (2020) found that college students changed their learning strategies to a more continuous habit during COVID-19 home confinements, which contributed to improved academic scores. Though that study focused only on changes of college students’ academic scores, the finding contrasted with the widely assumed learning loss concerns due to university lockdowns.

It needs to note that previous studies mostly investigated students’ changes of academic achievement due to the pandemic, impacts of university lockdowns on students’ other important learning outcomes have not been reported. One crucial learning outcome is critical thinking that is described as the acquisition of deep and meaningful understanding as well as critical inquiry skills and dispositions (Garrison et al., 2001). Critical thinking has been recognized as one of the most important learning outcomes expected of college students (Halpern, 1998; Shaw et al., 2020). Apparently, a comprehensive examination of students’ learning outcomes before and after the university lockdown is required to evaluate effects of the university lockdown upon both their academic achievement, as well as critical thinking skills and dispositions.

### Factors related to students’ learning during the lockdown period

In addition to possible changes in students’ learning outcomes, a more crucial issue is to clarify what might have shaped students’ post-lockdown learning outcomes given that the COVID-19 pandemic is an undeniably unique event with many new factors that may affect students’ learning. Though a thorough examination of all impacting factors is not possible, several of them are particularly notable in characterizing students’ at-home learning during the lockdown period due to COVID-19.

First, the outbreak of the coronavirus pandemic has pushed most universities toward distance education, which was probably the only option to mitigate the adverse influence of the disruption to classroom learning. Though few were ready for this abrupt transition, students’ skills and attitudes toward online learning are important factors to keep them actively engaged online (Bolliger, 2004; Hsu et al., 2019). Research shows that online learning successes rely heavily on students’ self-management skills (Wang, 2011; Broadbent, 2017; Panadero et al., 2019). Self-management skills include students’ ability to evaluate, monitor, and regulate themselves and assume responsibility for their own performance (Frayne, 1991; Gerhardt, 2007). These skills are especially valuable qualities for online learning at home given that many students may not have had practical guidelines they could follow during the pandemic.

Second, high rates of the transmission and mortality of COVID-19, combined with a lack of prevention and treatment measures, have left many people with cognitive uncertainty as well as negative emotions (Shanahan et al., 2020; Wang et al., 2020; Rodriguez et al., 2021). High rates of psychological distress have been reported among young adults in response to the pandemic (Xiong et al., 2020). Stressors such as fears of infection, frustration, and anger would likely have enduring effects on college students and their learning performance (Hasan and Bao, 2020). Thus, it is assumed students’ cognitive and emotional responses to the pandemic might affect their learning outcomes.

Third, research shows that the detrimental effects of school lockdown on K-12 students’ academic performance are particularly evident for students from disadvantaged family backgrounds (Kuhfeld et al., 2020; Parolin and Lee, 2021). Unlike typical learning activities conducted at universities, most college students learned from home during the pandemic. This means that college students’ learning is likely to be at least partially dependent upon their home environments and their daily family interactions. For example, college students are already adults and are expected to share domestic responsibilities with their parents when they stay at home. Thus, students’ family and home issues (e.g., taking care of family issues, dealing with housework chores) during the pandemic are likely to influence their time and effort put into learning, which may eventually affect their learning outcomes. Consequently, it is reasonable to assume that students’ time put into family or home responsibilities when they stayed at home might be another important type of factor influencing their post-lockdown learning outcomes.

### The present study

In this study, we examined impacts of COVID-19 university lockdown on college students’ learning outcomes including academic achievement and critical thinking, and further identified factors affecting their learning outcomes when students learned at home during the pandemic. The data used for analyses were drawn from a longitudinal study collecting data before and after the university lockdown from students at a public university in Zhengzhou, China, which is located approximately 3 h by train from Wuhan, the first epicenter of the global COVID-19 pandemic. The university, like most other universities in China, started the Lunar New Year vacation from the middle of January 2020, and shifted to distance education from February 2020 following China’s national school closure policy due to the outbreak of COVID-19 in late January 2020.^1^ Students were asked to study online and were not allowed to return to campus until September 2020. This meant that these students had stayed home for approximately 9 months (including a 2-month summer vacation). Our data were composed of pre- and post-lockdown assessments (with an interval of 12 months) of learning outcomes and several issues related to students’ learning performance. The unique feature of the data allowed us to evaluate changes (both performance average and variability) of students’ learning outcomes and to clarify factors influencing their post-lockdown outcomes when pre-pandemic levels were accounted for.

In addition, to examine changes of academic achievement and critical thinking, we evaluated a variety of learning-related factors including students’ readiness for online learning, their cognitive and emotional responses to the pandemic, time devoted to learning at home, and responsibilities at home to examine their potential effects on learning outcomes. Since family socioeconomic status (SES) has been shown to influence students’ educational outcomes (Sirin, 2005), we also measured students’ family SES as background variables. It was hypothesized that the learning-related factors would account for variations in students’ post-lockdown learning outcomes when their pre-lockdown outcomes and family SES were controlled for.

Eventually, this study aimed to clarify not only what has happened to college students’ learning during the COVID-19 lockdown period pandemic but also what might have shaped their academic achievement and critical thinking. Addressing both of these goals is crucial for higher education providers to deal with consequences of university closures during the COVID-19 pandemic and similar situations in the future.

## Materials and methods

### Sample and procedures

As stated earlier, data came from a longitudinal, ongoing research project on college students’ academic development. The project was initiated in October 2018, when 648 first-year college students were recruited via the university subject pool. Participants were informed that they would be contacted and tested again in October 2019, and October 2020. A new cohort of first-year students (*N* = 364) was recruited and added to the sample pool in October 2019, and was retested in October 2020. Students’ learning-related skills and dispositions were measured each year with scales and questionnaires varied slightly from year-to-year. This paper used the data collected in October 2019 as the pre-lockdown data (except for the critical thinking skill test, which was collected in October 2018), and used those collected in October 2020 as the post-lockdown data. This provided us with 1,004^2^ effective participants (635 women, *M* age = 21.67, *SD* age = 1.01 at October 2020). Approximately 80% of the sample majored in social science, with 20% in natural sciences and mathematics. In addition, 38% of the sample grew up in urban areas, 56% in rural areas, and 6% did not indicate the rural/urban information. The study protocol was approved by the Human Subjects Review Committee of the Huazhong University of Science and Technology. Each participant signed a written informed consent describing the study purpose, procedure and right of withdrawal during the study.

All measures were administered in a computer room at the university. Participants were tested in groups of 35–55 by two research assistants. Each participant completed the measures on a computer. The pre-lockdown testing included measures of socioeconomic status, perceived academic achievement, and critical thinking skills and dispositions. Besides the pre-lockdown measures, the post-lockdown testing additionally included questionnaires assessing students’ readiness for online learning, students’ cognitive and emotional responses to the pandemic, their time devoted to learning, and responsibilities at home when they stayed at home during the pandemic.

### Measures of learning outcomes

#### Grade point average

College grade point average (GPA) was gathered from student records. The GPA for the autumn semester of 2019 and the spring semester of 2020 were used as academic scores before and after the university lockdown. However, we learned from the university’s academic affairs division that exam difficulty in the spring semester of 2020 was set by many teachers at a relatively low level given that students had experienced a disruption to normal schooling by the pandemic. Therefore, GPA scores after the university lockdown (*M* = 3.42, *SD* = 0.51) were relatively high compared to those before the lockdown (*M* = 2.65, *SD* = 0.82). We thus employed a standard test to assess students’ self-perceived academic achievement (see below) as a supplement.

#### Perceived academic achievement

Perceived academic achievement was assessed by a subscale from the Student Learning and Development Scale (Wei et al., 2015), which was developed to assess Chinese undergraduate students’ academic, social, and practical development. The academic subscale was especially suitable for assessing perceived academic achievement of Chinese college students (Ren et al., 2020). The subscale has five items measuring multiple dimensions of students’ academic performance: students’ level of professional knowledge, students’ level of research methods in the subjects they have learned, students’ comprehensive applied ability, students’ level of reading and comprehension, and students’ ability of using information technology for learning and research. Participants were asked to indicate how much progress they had made in each of the academic dimensions over the past year. The subscale used a 4-point scale (1 = *no improvement*; 4 = *greatly improved*). Scores of this subscale ranged from 5 to 20. Higher scores reflected larger achievement. The subscale showed good reliabilities and construct validities among Chinese college students (Wei et al., 2015). The internal consistency reliability in the current study was 0.86.

#### Critical thinking skills

The Chinese Critical Thinking Test was used to tap students’ critical thinking skills (Luo and Yang, 2002). This test was designed based on the California Critical Thinking Skills Test (Facione et al., 2002). For the Chinese version, Luo and Yang generated test items according to lifestyles of Chinese students. The test includes 34 multiple-choice items, each of which has four response options, with only one being correct. The items assess five critical thinking skills: evaluation, inference, analysis, inductive reasoning, and deductive reasoning. The score was the total number of correctly answered items. As reported by Luo and Yang (2002), the test-retest correlation (1 month between time 1 and time 2) was 0.63, and the split-half reliability was 0.80. In addition, there were moderate to strong correlations between scores of the subscales and the total score (>0.50, Lin, 2018), supporting the construct validity of the test. The internal consistency reliability of this scale was 0.64 in the current study.

#### Critical thinking dispositions

The Chinese Critical Thinking Dispositions Inventory (Ren et al., 2020) was developed according to the conceptual framework of the California Critical Thinking Disposition Inventory (Facione et al., 2001). In this study, we used three subscales of the inventory to shorten testing time and reduce students’ fatigue. The three subscales assessed critical thinking dispositions (truth-seeking, analyticity and systematicity) that have been frequently studied in the critical thinking literature. Truth-seeking reflects one’s objectivity with findings even if the findings do not support one’s preconceived opinions. Analyticity refers to the disposition of applying reasoning and the use of evidence to solve problems. Systematicity reflects one’s disposition of being organized and orderly in inquiry. Each disposition dimension was measured by five items. Since the current research treats critical thinking dispositions as a component of critical thinking, the total score of the test was used. Participants rated items using a 6-point scale (1 = *strongly disagree*; 6 = *strongly agree*). The internal consistency reliability of this inventory was 0.82 based on data of our study.

### Measures of potential factors related to students’ learning

#### Readiness for online learning

This questionnaire was adapted from the online learning instrument developed by Smith et al. (2003) to assess students’ readiness for online learning. The Supplementary material presents all items of this measure, as well as items of other measures of potential factors associated with learning. The online readiness questionnaire assesses two important factors: the readiness for online learning skills, reflecting student’s comfort with basic online learning skills (e.g., computing, communication, and keyboarding), and the readiness for self-management learning, reflecting the readiness for organization, time-management, and independence necessary for online learning. The readiness for online learning skills factor included six items (e.g., “I was able to easily access the Internet as needed for my studies”). The readiness for self-management of learning factor included four items (e.g., “I was able to manage my study time effectively and easily completed assignments on time”). Students indicated their agreement using a 5-point scale (1 = *strongly disagree*; 5 = *strongly agree*). Separate scores were computed for the two subscales of online learning. Higher scores reflected better preparation for online learning. The questionnaire showed a good reliability (with a Cronbach alpha of 0.83), and yielded a two-factor structure that was readily interpretable in the framework of existing theory (Smith et al., 2003). Reliabilities of these subscales ranged from 0.77 to 0.83 in the current study.

#### Cognitive and emotional responses to COVID-19

This questionnaire was adapted from the psychological response questionnaire from the severe acute respiratory syndrome pandemic (Qian et al., 2005). The revised questionnaire included two subscales evaluating students’ cognitive and emotional responses toward COVID-19. The cognitive subscale included four items measuring the level of one’s awareness and control of the pandemic (e.g., “I thought that I should take all actions to avoid being infected by the virus”). The emotional subscale included four items assessing one’s levels of nervousness, anger, pessimism, and helplessness during the pandemic (e.g., “When I learned that a case was found in our city, I felt helpless”). Participants responded to each item using a 5-point scale (1 = *not at all typical of me*; 5 = *very typical of me*). Separate scores were computed for each subscale. Higher scores on the cognitive subscale reflected more reasonable responses to the pandemic, while higher scores on the emotional subscale reflected more negative responses. Internal consistency reliabilities of these subscales were both 0.74 in the current study.

#### Students’ time devoted to learning at home

This questionnaire evaluated students’ time devoted to learning at home during the pandemic. Prior to formal testing, we piloted the questionnaire with a small number of students to check for general understandability, and we made minor revisions based on their feedback. The questionnaire included three items covering the following aspects: students’ time spent on online learning, time used for completing the study plan; and time that was not interrupted for learning (see all the items in the Supplementary material). Students rated each item using a 5-point scale (1 = *strongly disagree*; 5 = strongly agree). We computed a total score by summing scores of the items. Higher scores reflected more time students were able to devote to learning at home during the lockdown period. The internal consistency reliability of this questionnaire was 0.63 in the current study.

#### Students’ responsibilities at home during the lockdown

This questionnaire was developed to evaluate the degree of students’ responsibilities when they stayed at home during the lockdown. There were three items each covering the following responsibilities: taking care of family members, doing housework, and dealing with home affairs. Each item was rated using a 5-point scale (1 = *strongly disagree*, 5 = *strongly agree*). The higher the score, the higher level of responsibilities at home. In the current study, the internal consistency reliability of this questionnaire was 0.80.

### Measures of socioeconomic status

Data of students’ family SES were collected and used as controlling variables in examining associations of the learning-related factors with the learning outcomes. We used monthly household income, parents’ occupation, and parents’ level of education to estimate SES. Family household income was measured with a 10-point scale (1 = less than 2,000, 2 = 2,001–3,000, 3 = 3,001–4,000, 4 = 4,001–5,000, 5 = 5,001–6,000, 6 = 6,001–7,000, 7 = 7,001–8,000, 8 = 8,001–9,000, 9 = 9,001–10,000, and 10 = more than 10,000 Chinese Yuan per month). Both parents’ occupations were measured using the Occupational Prestige Scale (Lu, 2003), in which contemporary Chinese occupations were rated according to the status of ownership of organizational, economic and cultural resources (1 = unemployed, 2 = agricultural laborer, 3 = industrial workers, 4 = business service employees, 5 = industrial and commercial households, 6 = clerks, 7 = professional and technical personnel, 8 = private businessman, 9 = managers, and 10 = state and social managers). Higher scores represented higher prestige of an occupation. Each parent’s educational level was assigned a value from 1 to 6 (1 = elementary school and below, 2 = junior high school, 3 = high school or technical secondary school, 4 = junior college, 5 = bachelor, and 6 = master and above).

### Data cleaning

First, we checked the datasets for the number of participants for each variable. The valid number of participants was 1,004 except for GPA, perceived academic achievement, and critical thinking skills. GPA scores were available for 853 students in the pre-lockdown testing, and 868 in the post-lockdown testing. Data of perceived academic achievement were available for 642 students (because only the 2018 cohort completed this test, and the 2019 cohort did not shorten the overall survey). Data of critical thinking skills were available for 642 students (only the 2018 cohort completed this test). Second, we performed analyses to detect outliers. Any observation exceeding three standard deviations from the means was replaced with a value that was three standard deviations. This procedure affected no more than 5% of observations.

### Analytic strategy

First, we performed tests of measurement invariance for the learning outcome measures (perceived academic performance, critical thinking skills, and critical thinking dispositions) across the time points before and after the university lockdown. Following established procedures (Putnick and Bornstein, 2016), we compared the configural invariance model (i.e., models had the same pattern of factor structure, but factor loadings between models were allowed to vary across times), the metric invariance model in which factor loadings were constrained to be the same across times, and the scalar invariance model in both factor loadings and intercepts were constrained to be the same across times). Each model was evaluated using the *χ^2^*, comparative fit index (CFI), the root-mean-square error of approximation (RMSEA) by means of LISREL 8.7. Metric invariance was supported if the metric model did not result in significant changes in model fit compared to the configural model (ΔCFI ≤ 0.01, and ΔRMSEA ≤ 0.015 suggest no substantial change according to Chen, 2007). Likewise, scalar invariance was supported if the scalar model did not significantly degrade relative to the metric model.

Paired sample *t*-tests were first performed to compare the average performance of the learning outcomes (except GPA) before and after the university lockdown. To evaluate changes of the variability of the learning outcomes, we computed the coefficient of variation (CV = SD/mean) for each learning outcome. The CV is a statistical measure of the variability of a distribution of repeated measurements or a data set. As a relative difference quantity, the CV can be used not only for comparing variability across repeated measurements, but also for comparing variability across sets of measurements on different scales (Shechtman, 2013). A larger CV value reflects greater variability.

Next, following the procedure described by Shanahan et al. (2020), regression analyses were performed in separate steps to examine relationships between the pre-lockdown learning outcomes and the proposed learning-related factors with the post-lockdown learning outcomes. First, we examined the association between each of the learning-related factors and each of the post-lockdown learning outcomes by controlling for SES and the corresponding prior learning outcome. This was achieved by performing a hierarchical linear regression with SES and the prior learning outcome in the first step, and a learning-related factor in the second step. This step was repeated for each post-lockdown learning outcome and each learning-related factor. By doing this, we obtained the association between each learning-related factor and each learning outcome after adjusting for SES and the prior learning outcome. Second, all the significant learning-related factors from the first step were entered into one regression model for predicting each post-lockdown learning outcome. SES and the prior learning outcome served as control variables. This step was repeated for all learning outcomes. The second step resulted in trimmed models with final SES factors, the prior learning outcomes, and learning-related factors related as predictors of post-lockdown outcomes.

## Results

Table 1 presents the samples, means, and standard deviations for each of the variables. The intercorrelations among the variables at both time points were presented in Supplementary Table 2. Results indicated longitudinal metric invariance (ΔCFI = −0.001 ≤ 0.01, and ΔRMSEA = −0.002 ≤ 0.015, for details of the model fit see Supplementary Table 3), and scaler invariance (ΔCFI = 0.002 ≤ 0.01, and ΔRMSEA = −0.005 ≤ 0.015) across the time points before and after the lockdown. The establishment of the measurement invariance allowed us to directly compare scores of the learning outcomes across the two times and examine relations of the study variables.

**TABLE 1:** Descriptive statistics for all variables of the study.

Variables	*N*	*M*	*SD*
**Socioeconomic status**			
Household income level	1,004	5.04	2.71
Father’s occupation level	1,004	4.71	2.71
Mother’s occupation level	1,004	3.89	2.64
Father’s educational level	1,004	2.76	1.21
Mother’s educational level	1,004	2.57	1.25
**Pre-lockdown learning outcomes**			
Grade point average	853	2.65	0.82
Perceived academic achievement	642	12.30	2.67
Critical thinking skills	642	20.47	3.26
Critical thinking dispositions	1,004	64.70	8.71
**Post-lockdown learning outcomes**			
Grade point average	868	3.42	0.51
Perceived academic achievement	642	12.21	2.78
Critical thinking skills	642	18.80	4.78
Critical thinking dispositions	1,004	63.14	9.05
**Potential learning-related factors**			
Time devoted to learning at home	1,004	10.86	2.16
Responsibilities at home	1,004	6.80	2.49
Cognitive response to the pandemic	1,004	12.68	3.53
Emotional response to the pandemic	1,004	10.45	3.23
Readiness for online learning skills	1,004	22.57	3.58
Readiness for self-management learning	1,004	14.00	2.92

**FIGURE 1:**
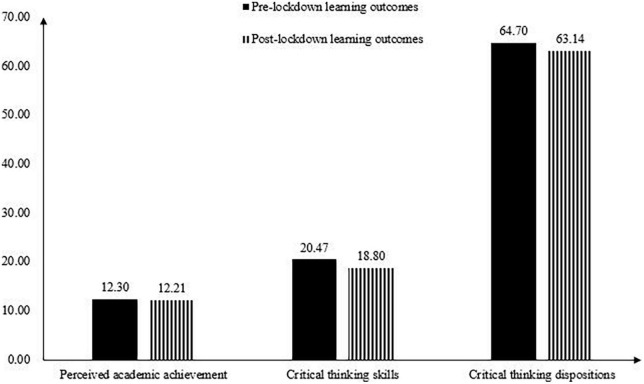
Illustration the average performance for each learning outcome before and after the university lockdown.

**FIGURE 2:**
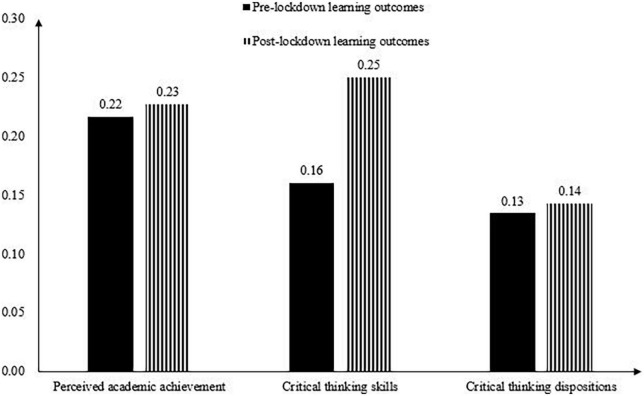
Illustrations of variability of each learning outcome before and after the university lockdown.

Figure 1 illustrates changes of the average performance for each learning outcome before and after the university lockdown (the GPA scores were not compared since, as mentioned earlier, the exam difficulty in 2020 was set at a relatively low level). Average scores of the learning outcomes showed an overall decreasing trend from pre- to post-lockdown. Paired sample *t-*tests revealed that critical thinking skills (*t* = 4.02, *p* < 0.01, Cohen’s *d* = 0.16), and critical thinking dispositions (*t* = 5.33, *p* < 0.01, Cohen’s *d* = 0.17) were lower after the university lockdown compared to their pre-lockdown assessments. No significant difference was found between the pre- and post- lockdown perceived academic achievement (*t* = 0.71, *p* > 0.05, Cohen’s *d* = 0.03). Figure 2 illustrates changes of the CVs for each learning outcome. It is evident that variability for each learning outcome showed an overall increasing trend from pre- to post-lockdown. Variability of the critical thinking skills showed a particularly large increase.

**FIGURE 3:**
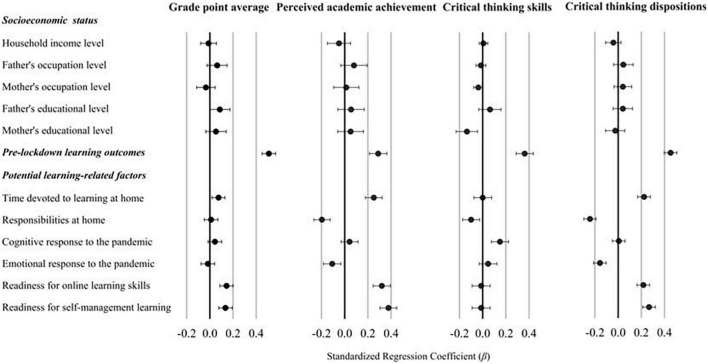
Associations of socioeconomic status (SES) and pre-lockdown learning outcomes with post-lockdown outcomes. Separated hierarchical linear regressions were conducted with SES factors and pre-lockdown learning outcomes in the first step, and each learning-related factor in the second step (i.e., a separate model for each learning-related factor). Standardized regression coefficients (β) and 95% CIs were applied.

Figure 3 presents the associations between each learning-related factor and post-lockdown learning outcome produced by regression analyses, after controlling for SES variables and pre-lockdown learning outcomes (for exact coefficients and *p*-values, see the Supplementary Table 4). As expected, prior outcome variables showed the largest associations with their corresponding post-lockdown outcomes. With respect to the proposed learning-related factors, students’ time devoted to learning was positively associated with GPA, perceived academic achievement and critical thinking dispositions, whereas students’ responsibilities at home were negatively associated with all outcome variables except GPA. Students’ cognitive response to the pandemic was positively related to critical thinking skills, while the emotional response was negatively associated with perceived academic achievement and critical thinking dispositions. Readiness for online learning skills and self-management learning were positively associated with all outcome variables except critical thinking skills.

Table 2 presents associations from the final trimmed models aiming to understand which factors explained unique variance of the post-lockdown outcomes after controlling for pre-lockdown learning outcomes and SES factors. The upper part of Table 2 includes all significant prior outcome variables and SES factors, and the lower part presents all the significant learning-related factors. Results of factors with *p* > 0.10 were not presented. The significant predictors are ordered by the size of the standardized regression coefficients. As shown in Table 2, readiness for self-management learning showed the largest effects on perceived academic achievement and critical thinking dispositions. Readiness for online learning skills showed consistently positive effects on all outcome variables except critical thinking skills. While students’ cognitive responses to the pandemic were positively related to critical thinking skills, their emotional responses were negatively associated with critical thinking dispositions. In addition, responsibilities at home were consistently associated with all outcome variables except GPA.

**TABLE 2:** Standardized regression coefficients from the final trimmed hierarchical linear regression models with socioeconomic status (SES) factors and pre-lockdown learning outcomes in the first step, and the potential learning-related factors that were significantly identified by the separated models as the second step.

Grade point average	Perceived academic achievement	Critical thinking skills	Critical thinking dispositions
SES factors and pre-lockdown learning outcomes	SES factors and pre-lockdown learning outcomes	SES factors and pre-lockdown learning outcomes	SES factors and pre-lockdown learning outcomes
• Grade point average (β = 0.52, *p* < 0.01)	• Perceived academic achievement (β = 0.18, *p* < 0.01)	• Critical thinking skills (β = 0.35, *p* < 0.01)	• Critical thinking dispositions (β = 0.37, *p* < 0.01)
		• Mother’s educational level (β = −0.14, *p* < 0.05)	
Factors related to learning during the pandemic	Factors related to learning during the pandemic	Factors related to learning during the pandemic	Factors related to learning during the pandemic
• Readiness for online learning skills (β = 0.10, *p* < 0.01)	• Readiness for self-management learning (β = 0.26, *p* < 0.01)	• Cognitive response to the pandemic (β = 0.15, *p* < 0.01)	• Readiness for self-management learning (β = 0.16, *p* < 0.01)
• Readiness for self-management learning (β = 0.08, *p* < 0.05)	• Readiness for online learning skills (β = 0.13, *p* < 0.01)	• Responsibilities at home (β = −0.11, *p* < 0.01)	• Responsibilities at home (β = −0.16, *p* < 0.01)
	• Responsibilities at home (β = −0.11, *p* < 0.01)		• Emotional response to the pandemic (β = −0.12, *p* < 0.01)
	• Emotional response to the pandemic (β = −0.08, *p* < 0.05)		• Time devoted to learning at home (β = 0.08, *p* < 0.05)
			• Readiness for online learning skills (β = 0.06, *p* = 0.10)

## Discussion

After the onset of the COVID-19 pandemic, many universities turned to distance education to limit viral transmission of infection, which influenced millions of college students, forcing them to adapt to new learning environments as well as ways of learning they may have been unaccustomed to. Such unexpected changes led to significant uncertainty in the learning and academic development of students (Conceição and Howles, 2020). Concerns have been raised regarding the consequences of school lockdowns on students’ academic development (e.g., Kuhfeld et al., 2020; Kaffenberger, 2021), and the consequences are not likely to fade as things return to “normal.” Unfortunately, empirical evidence has been limited in evaluating how university lockdowns during the pandemic impact students’ learning outcomes. In addition, the transition to online learning at home may lead to important factors radically different from those related to students’ learning during typical academic years. Clarifying these factors and their associations with students’ learning outcomes is crucial for higher education to develop learning-centered programs, and to validate non-standard or alternative learning formats for managing the ongoing pandemic crisis.

This study leveraged a longitudinal design to describe the average performance and variability of college students’ learning outcomes after they participated in online learning from home for an extended period Comparisons of students’ learning outcomes showed that while students’ perceived academic achievement showed no improvement, their critical thinking (both skills and dispositions) decreased significantly from pre- to post-lockdown. In addition, outcome variables after the lockdown exhibited overall greater variability relative to those prior to the pandemic, with critical thinking skills showing a particularly large increase. These results concur with the learning loss concerns identified by other education researchers (e.g., Kaffenberger, 2021), conveying worrying messages that college students learned less due to the disruption of traditional instruction. Moreover, the result that variability for each learning outcome showed an increasing trend suggests that the university lockdown was likely to widen variations in students’ learning outcomes.

Among the factors proposed to influence students’ learning, readiness for online learning was consistently associated with post-lockdown learning outcomes including academic achievement and critical thinking dispositions, even after controlling for pre-lockdown learning outcomes. The role of self-management learning was particularly evident in students’ self-perceived academic achievement and critical thinking dispositions. These results are consistent with previous work indicating that self-management learning affects students’ academic achievement and e-learning performance (Wang, 2011; Broadbent, 2017; Panadero et al., 2019). Self-management involves self-assessment, goal setting, time management, and regulating goal progress and attainment by using reinforcement and punishment (Frayne, 1991), which is recognized as a prerequisite for effective learning in distance education (Evans, 2000). Indeed, the need for self-management learning runs clearly throughout the distant learning literature, and in most online learning delivery formats (Bernard et al., 2004).

Compared with students’ time devoted to learning at home, their responsibilities at home exhibited relatively large effects on learning outcomes: those who assumed more responsibilities at home during the pandemic reported lower scores on perceived academic achievement, critical thinking skills and dispositions. As stated earlier, college students are already adults who are expected to share household responsibilities with their parents when they stay at home. Greater responsibilities at home may put considerable pressure on students’ learning behaviors especially given that they were experiencing disruptions to the location and format of their schooling prior to the lockdown. It is possible that students’ added responsibilities at home, coupled with the sudden shift to online learning, created a challenging learning environment making them feel “out of control” or at least less in control than normal.

Interestingly, whereas students’ cognitive responses to the pandemic were positively related to critical thinking skills, their emotional responses were negatively related to critical thinking dispositions. Note that these findings were observed even after adjusting for students’ pre-pandemic critical thinking skills/dispositions. Though the reported associations were only weak to moderate, they did reveal the possibility that students’ psychological responses to the pandemic may alter their higher-order learning outcomes. A reasonable perception of and response to the pandemic may serve as a conductive buffer layer for one to analyze and understand new knowledge. In contrast, negative emotional responses experienced due to an awareness of the possible consequences of the pandemic may to some extent affect one’s willingness or ability to apply critical thinking.

### Limitations

Our study has the strength of including both pre- and post-lockdown assessments of both students’ academic achievement and critical thinking. However, it also faces a few limitations. First, the sample is potentially not representative of students from other kinds of institutions (e.g., those from other countries, of different sizes, or private compared to public). While the lockdown policy in China was virtually identical across higher educational institutions, the homogeneity of the sample may also affect the generalizability of our results to nations or regions with different lockdown strategies, different rates of the pandemic, and different social or educational systems.

Second, though the pre-pandemic learning outcomes were controlled in examining associations between the proposed learning-related issues during the pandemic and post-lockdown learning outcomes, the reported associations are correlational in nature, and cannot be given direct causal interpretations.

A third limitation is that our assessment of the learning-related factors took place immediately after the lockdown ended. Students’ responses to some factors (e.g., emotional responses during the pandemic) might to some extent be distorted by the prolonged lockdown. Fourth, the pre-lockdown assessment occurred approximately 3 months before the outbreak of COVID-19 (the critical thinking skills were measured around 15 months before the outbreak). It is possible that some of the observed changes in learning outcomes may partly result from factors preceding the pandemic.

### Implications

Despite the limitations, our findings have important implications for college students’ educational development as well as for educational practices when lockdown measures are imposed during a severe pandemic like COVID-19. First, decreases in critical thinking are especially disquieting since it plays a crucial role in students’ later academic achievement and even their future professional development (Shaw et al., 2020). This calls for a consideration of corrective measures to maximize the recovery of learning losses. For example, instructions that have been demonstrated effective for improving college students’ critical thinking (e.g., Heijltjes et al., 2014) might be referenced to develop special programs for promoting their critical thinking skills.

Second, given the significant roles of self-management in students’ learning outcomes, higher education institutions might prepare students with necessary and important skills (e.g., evaluating, monitoring, and regulating oneself) for online learning via tutorials of self-management training. Third, during times of unexpected disruption to normal schooling, universities and departments should provide supportive measures such as providing attractive learning materials or secure internet access (Hasan and Bao, 2020), and educate students about coping strategies such as engaging in physical exercise, or positive reappraisal (Shanahan et al., 2020) to alleviate their emotional distress and counteract the adverse effects on students’ learning performance.

## Conclusion

Our study assessed college students’ academic achievement and critical thinking before and after the COVID-19 university lockdown. We also assessed several factors hypothesized to influence students’ learning from home during the lockdown period. We observed significant decreases in critical thinking skills and dispositions among students from the pre- to post-lockdown, and both achievement and critical thinking exhibited overall greater variability after the lockdown. In addition, students’ readiness for online learning, especially their self-management skills, consistently predicted post-lockdown academic achievement and critical thinking after pre-lockdown outcomes and family SES were controlled for. Those who assumed more responsibilities at home, or who were more vulnerable to emotional distress during the pandemic, performed less well in post-lockdown learning outcomes. These findings call for better management of student learning and development when major changes are required in higher education practices for responding to the ongoing COVID-19 crisis as well as other potential situations.

## Data availability statement

The raw data supporting the conclusions of this article will be made available by the authors, without undue reservation.

## Ethics statement

The studies involving human participants were reviewed and approved by the Human Subjects Review Committee of the Huazhong University of Science and Technology. The participants provided their written informed consent to participate in this study.

## Author contributions

XL and JM collected the data and wrote the manuscript. SZ assisted in data collection. TB assisted in re-drafting and editing the manuscript. XR designed the study and wrote the manuscript. All authors contributed to the article and approved the submitted version.

## Footnotes

1 In late January 2020, China postponed all educational activities and the Chinese Ministry of Education urged higher education institutions to use online delivery as an alternative to face-to-face teaching. In response to the call, higher education institutions in mainland China transitioned to online teaching for the spring semester of 2020. College students in most universities were not allowed to return to campus until the start of the autumn semester of 2020.

2 Eight participants were omitted from the post-lockdown survey and their data were not included.
