# Supplementary material for: Impacts of university lockdown during the coronavirus pandemic on college students’ academic achievement and critical thinking: A longitudinal study
Source: Front Psychol. 2022 Oct 26;13:995784. doi: 10.3389/fpsyg.2022.995784 (PMC9643715; doi:10.3389/fpsyg.2022.995784)
Supplement: Supplementary file 1 [file Table_1.DOCX]

**Supplementary materials**

| **Table 1**. Questionnaire items of the learning-related factors of the study. Relevant citations can be found in the measures section of the article. | |
| --- | --- |
| **Measure** | **Item(s)** |
| Time devoted to learning at home | - I had sufficient time for conducting online learning at home during the pandemic. - I had plenty of time to complete my study plan during the pandemic. - I had plenty of time to study without being disturbed by family members during the pandemic. |
| Responsibilities at home | - I spent a lot of effort taking care of my family during the pandemic. - I was responsible for a lot of housework at home during the pandemic. - I had to be involved and deal with a lot of home affairs during the pandemic. |
| Cognitive responses to the COVID-19 | - I thought that anyone might be a COVID-19 case. - I thought that my health might be threatened by COVID-19. - I thought that my life might be threatened by COVID-19. - I thought that I should take all actions that I can do to avoid being infected with COVID-19. |
| Emotional responses to the COVID-19 | - When I learned that a COVID-19 case had been found in our city, I felt nervous. - When I learned that a COVID-19 case had been found in our city, I felt angry. - When I learned that a COVID-19 case had been found in our city, I felt pessimistic. - When I learned that a COVID-19 case had been found in our city, I felt helpless. |
| Readiness for online learning skills | - I was able to easily access the Internet as needed for my studies. - I was comfortable communicating electronically. - I was willing to actively communicate with my classmates and instructors electronically. - I felt that my background and experience were sufficient for my online studies. - I possessed sufficient computer keyboarding skills for doing online work. - I felt comfortable with communicating online. |
| Readiness for self-management learning | - When it came to learning and studying, I was a self-directed person. - In my studies, I was self-disciplined and found it easy to set aside reading and homework time. - I was able to manage my study time effectively and easily completed assignments on time. - In my studies, I set goals and had a high degree of initiative. |

| **Table 2**. The intercorrelations among the variables at both time points. | | | | | | | | | | | | | | | | | | |
| --- | --- | --- | --- | --- | --- | --- | --- | --- | --- | --- | --- | --- | --- | --- | --- | --- | --- | --- |
|  | 1 | 2 | 3 | 4 | 5 | 6 | 7 | 8 | 9 | 10 | 11 | 12 | 13 | 14 | 15 | 16 | 17 | 18 |
| ***Socioeconomic status*** |  |  |  |  |  |  |  |  |  |  |  |  |  |  |  |  |  |  |
| 1. Household income level | _ |  |  |  |  |  |  |  |  |  |  |  |  |  |  |  |  |  |
| 2. Father’s occupation level | 0.55^**^ | _ |  |  |  |  |  |  |  |  |  |  |  |  |  |  |  |  |
| 3. Mother’s occupation level | 0.48^**^ | 0.64^**^ | _ |  |  |  |  |  |  |  |  |  |  |  |  |  |  |  |
| 4. Father’s educational level | 0.37^**^ | 0.60^**^ | 0.51^**^ | _ |  |  |  |  |  |  |  |  |  |  |  |  |  |  |
| 5. Mother’s educational level | 0.36^**^ | 0.55^**^ | 0.56^**^ | 0.71^**^ | _ |  |  |  |  |  |  |  |  |  |  |  |  |  |
| ***Pre-lockdown Learning outcomes*** |  |  |  |  |  |  |  |  |  |  |  |  |  |  |  |  |  |  |
| 6. Grade point average | -0.06 | -0.10^**^ | -0.11^**^ | -0.08^*^ | -0.13^**^ | _ |  |  |  |  |  |  |  |  |  |  |  |  |
| 7. Perceived academic achievement | 0.11^**^ | 0.10^**^ | 0.13^**^ | 0.14^**^ | 0.13^**^ | -0.05 | _ |  |  |  |  |  |  |  |  |  |  |  |
| 8. Critical thinking skills | 0.00 | -0.05 | -0.06 | 0.01 | 0.00 | 0.04 | -0.04 | _ |  |  |  |  |  |  |  |  |  |  |
| 9. Critical thinking dispositions | 0.09^**^ | 0.13^**^ | 0.11^**^ | 0.09^**^ | 0.11^**^ | 0.04 | 0.36^**^ | 0.03 | _ |  |  |  |  |  |  |  |  |  |
| ***Post-lockdown learning outcomes*** |  |  |  |  |  |  |  |  |  |  |  |  |  |  |  |  |  |  |
| 10. Grade point average | 0.03 | 0.06 | 0.01 | 0.09^*^ | 0.04 | 0.49^**^ | 0.13^**^ | 0.04 | 0.10^**^ | _ |  |  |  |  |  |  |  |  |
| 11. Perceived academic achievement | 0.08^*^ | 0.15^**^ | 0.14^**^ | 0.17^**^ | 0.16^**^ | -0.03 | 0.31^**^ | 0.01 | 0.20^**^ | 0.10^*^ | _ |  |  |  |  |  |  |  |
| 12. Critical thinking skills | -0.05 | -0.09^*^ | -0.11^**^ | -0.06 | -0.12^**^ | 0.16^**^ | -0.09^*^ | 0.37^**^ | 0.01 | 0.06 | -0.02 | _ |  |  |  |  |  |  |
| 13. Critical thinking dispositions | 0.05 | 0.12^**^ | 0.11^**^ | 0.10^**^ | 0.09^**^ | 0.07 | 0.21^**^ | 0.13^**^ | 0.46^**^ | 0.10^**^ | 0.39^**^ | 0.18^**^ | _ |  |  |  |  |  |
| ***Potential learning-related factors*** |  |  |  |  |  |  |  |  |  |  |  |  |  |  |  |  |  |  |
| 14. Time devoted to learning at home | 0.08^**^ | 0.11^**^ | 0.12^**^ | 0.16^**^ | 0.17^**^ | -0.02 | 0.20^**^ | 0.06 | 0.16^**^ | 0.09^**^ | 0.32^**^ | 0.00 | 0.29^**^ | _ |  |  |  |  |
| 15. Responsibilities at home | -0.03 | -0.11^**^ | -0.05 | -0.11^**^ | -0.10^**^ | -0.09^**^ | -0.04 | -0.06 | -0.09^**^ | -0.05 | -0.22^**^ | -0.10^**^ | -0.28^**^ | -0.22^**^ | _ |  |  |  |
| 16. Cognitive response to the pandemic | 0.02 | 0.01 | -0.01 | 0.03 | 0.00 | -0.04 | -0.02 | 0.03 | -0.03 | 0.04 | 0.03 | 0.17^**^ | -0.01 | 0.09^**^ | 0.09^**^ | _ |  |  |
| 17. Emotional response to the pandemic | 0.04 | -0.01 | -0.04 | -0.01 | -0.01 | 0.01 | -0.06 | -0.05 | -0.11^**^ | 0.00 | -0.13^**^ | 0.04 | -0.21^**^ | -0.03 | 0.25^**^ | 0.46^**^ | _ |  |
| 18. Readiness for online learning skills | 0.07^*^ | 0.12^**^ | 0.13^**^ | 0.14^**^ | 0.10^**^ | -0.09^**^ | 0.20^**^ | 0.04 | 0.11^**^ | 0.11^**^ | 0.38^**^ | -0.01 | 0.27^**^ | 0.47^**^ | -0.21^**^ | 0.11^**^ | -0.02 | _ |
| 19. Readiness for self-management learning | 0.08^*^ | 0.16^**^ | 0.16^**^ | 0.18^**^ | 0.18^**^ | 0.00 | 0.30^**^ | -0.07 | 0.26^**^ | 0.16^**^ | 0.45^**^ | -0.10^*^ | 0.37^**^ | 0.56^**^ | -0.21^**^ | 0.07^*^ | 0.00 | 0.62^**^ |

| **Table 3.** Fit indices of the measurement invariance models including the perceived academic achievement, critical thinking skills, and critical thinking dispositions as latent variables, and respective dimensions as indicators. | | | | | |
| --- | --- | --- | --- | --- | --- |
| Model | χ2_(df)_ | CFI | ΔCFI | RMSEA (90% CI) | ΔRMSEA |
| Configural | 236.087 (82) | 0.974 | _ | 0.057 (0.049-0.065) | _ |
| Metric | 267.406 (90) | 0.973 | -0.001 | 0.056 (0.048-0.063) | -0.002 |
| Scalar | 267.406 (101) | 0.975 | 0.002 | 0.051 (0.043-0.058) | -0.005 |
| Note. *N* = 642. | | | | | |

| **Table 4**. The association between each of the learning-related factors and each post-lockdown learning was outcome produced by regression analyses, after controlling for SES variables and prior learning outcomes during COVID-19 as displayed in Figure 3 of the article. Learning-related factors each entered one at a time (i.e., each learning factor = a separate model). This table presents all standardized regression coefficients (*β*) and 95% CIs from the regression models. | | | | | | | | | | | | |
| --- | --- | --- | --- | --- | --- | --- | --- | --- | --- | --- | --- | --- |
|  | **Grade point average** | | | **Perceived academic achievement** | | | **Critical thinking skills** | | | **Critical thinking dispositions** | | |
|  | *β* | 95% CI | *p* | *β* | 95% CI | *p* | *β* | 95% CI | *p* | *β* | 95% CI | *p* |
| ***Socioeconomic status*** |  |  |  |  |  |  |  |  |  |  |  |  |
| Household income level | -0.01 | -0.08 to 0.06 | 0.76 | -0.05 | -0.15 to 0.05 | 0.30 | 0.01 | -0.03 to 0.04 | 0.88 | -0.04 | -0.11 to 0.03 | 0.24 |
| Father’s occupation level | 0.06 | -0.02 to 0.15 | 0.15 | 0.08 | -0.03 to 0.19 | 0.17 | -0.02 | -0.06 to 0.03 | 0.80 | 0.05 | -0.04 to 0.13 | 0.29 |
| Mother’s occupation level | -0.03 | -0.11 to 0.05 | 0.40 | 0.01 | -0.09 to 0.12 | 0.80 | -0.04 | -0.08 to 0.00 | 0.49 | 0.04 | -0.04 to 0.12 | 0.30 |
| Father’s educational level | **0.09** | 0.00 to 0.17 | **0.05** | 0.06 | -0.06 to 0.17 | 0.35 | 0.06 | -0.03 to 0.16 | 0.28 | 0.04 | -0.04 to 0.13 | 0.34 |
| Mother’s educational level | 0.05 | -0.04 to 0.14 | 0.22 | 0.05 | -0.06 to 0.16 | 0.37 | **-0.14** | -0.23 to -0.04 | **0.02** | -0.03 | -0.11 to 0.06 | 0.56 |
| ***Pre-lockdown learning outcomes*** | **0.51** | 0.45 to 0.57 | **0.00** | **0.29** | 0.21 to 0.36 | **0.00** | **0.36** | 0.29 to 0.44 | **0.00** | **0.45** | 0.40 to 0.51 | **0.00** |
| ***Potential learning-related factors*** |  |  |  |  |  |  |  |  |  |  |  |  |
| Time devoted to learning at home | **0.08** | 0.02 to 0.13 | **0.01** | **0.25** | 0.18 to 0.32 | **0.00** | 0.00 | -0.08 to 0.08 | 0.99 | **0.22** | 0.17 to 0.28 | **0.00** |
| Responsibilities at home | 0.01 | -0.05 to 0.07 | 0.70 | **-0.20** | -0.27 to -0.13 | **0.00** | **-0.10** | -0.17 to -0.03 | **0.01** | **-0.24** | -0.30 to -0.19 | **0.00** |
| Cognitive response to the pandemic | 0.04 | -0.01 to 0.10 | 0.14 | 0.04 | -0.03 to 0.11 | 0.26 | **0.15** | 0.07 to 0.22 | **0.00** | 0.01 | -0.05 to 0.06 | 0.84 |
| Emotional response to the pandemic | -0.02 | -0.08 to 0.04 | 0.58 | **-0.11** | -0.18 to -0.03 | **0.00** | 0.05 | -0.03 to 0.12 | 0.20 | **-0.16** | -0.21 to -0.10 | **0.00** |
| Readiness for online learning skills | **0.14** | 0.09 to 0.20 | **0.00** | **0.32** | 0.25 to 0.39 | **0.00** | -0.01 | -0.09 to 0.07 | 0.74 | **0.22** | 0.16 to 0.27 | **0.00** |
| Readiness for self-management learning | **0.14** | 0.08 to 0.19 | **0.00** | **0.38** | 0.30 to 0.45 | **0.00** | -0.01 | -0.09 to 0.07 | 0.18 | **0.27** | 0.21 to 0.32 | **0.00** |
| *Note.* Coefficients with *p* < 0.05 in bold. | | | | | | | | | | | | |
